# Synthesis and Evaluation of Clove Oil‐Loaded Solid Lipid Nanoparticles on *Echinococcus granulosus* Protoscoleces

**DOI:** 10.1155/japr/3101296

**Published:** 2026-09-10

**Authors:** Niloufar Mansouri, Zahra Hesari, Sara Nemati, Mohammad Zibaei, Ehsan Javanmard, Zahra Mirabedini, Abolfazl Miahipour, Hamed Mirjalali

**Affiliations:** ^1^ Department of Medical Parasitology and Mycology, School of Medicine, Alborz University of Medical Sciences, Karaj, Iran, abzums.ac.ir; ^2^ Department of Pharmaceutics, School of Pharmacy, Guilan University of Medical Sciences, Rasht, Iran, gums.ac.ir; ^3^ Foodborne and Waterborne Diseases Research Center, Research Institute for Gastroenterology and Liver Diseases, Shahid Beheshti University of Medical Sciences, Tehran, Iran, sbmu.ac.ir; ^4^ Department of Parasitology and Mycology, School of Medicine, Shiraz University of Medical Sciences, Shiraz, Iran, sums.ac.ir; ^5^ Department of Medical Parasitology and Mycology, School of Public Health, Tehran University of Medical Sciences, Tehran, Iran, tums.ac.ir

**Keywords:** cell toxicity, clove oil extract, cystic echinococcosis, solid lipid nanoparticles

## Abstract

The most prevalent approach during treatment of cystic echinococcosis (CE) relies on surgical cyst removal and long‐term chemotherapy with benzimidazoles. However, cyst rupture may lead to anaphylaxis or secondary cysts and adverse effects. This study is aimed at synthesizing and evaluating the potential of clove oil‐loaded solid lipid nanoparticles (ClO‐SLNs) against *Echinococcus granulosus* protoscoleces. Clove bud (*Syzygium aromaticum*) oil was purchased and ClO‐SLNs were synthesized. Physicochemical characterization of the nanoparticles was performed using transmission electron microscopy (TEM) and their zeta potential was measured. In addition, encapsulation efficiency (EE%) and drug loading (DL%) were performed. The cytotoxicity of different concentrations of ClO‐SLNs was evaluated on HepG2 cell line. Protoscolicidal effects of different concentrations of ClO‐SLNs were investigated as well. One‐way ANOVA followed by post hoc tests where appropriate was employed to analyze data. Nanoparticles with round smooth margins were characterized by TEM. The size of particles was about 122.3 ± 30.0 nm with a zeta potential of −0.9 mV. The EE% and DL were 59.68% and 16.08%, respectively. The cell viability remained high (> 80%) across two concentrations, 100 *μ*g/mL and 1 mg/mL, of ClO‐SLNs. Raw clove oil extract exhibited no cytotoxicity at 50 mg/mL and the cell viability of albendazole was more than 60% at 10 mg/mL. The mortality rates of protoscoleces exposed to ClO‐SLNs were concentration‐dependent in all time points with *p* values: 0.013, 0.0074, 0.0064, and 0.0042 for 15, 30, 60, and 120 min. The ClO‐SLNs exhibit good biocompatibility across all tested concentrations with highly potent and rapid protoscolicidal activity against *E. granulosus* protoscoleces. These findings suggest that ClO‐SLNs may represent a potential protoscolicidal candidate for future intraoperative applications. However, further in vivo studies are required before considering clinical evaluation.

## 1. Introduction

ystic echinococcosis (CE; hydatid disease) is a major neglected zoonotic parasitic infection caused by the larval stage of *Echinococcus granulosus*, with humans becoming infected as accidental intermediate hosts through ingestion of eggs from dog feces [1, 2]. The disease affects more than 1 million people, mostly reported from South America, East Africa, Central Asia, and parts of China [3].

Standard management of CE relies on surgical cyst removal (often combined with the PAIR technique: puncture, aspiration, injection, reaspiration) for uncomplicated hepatic cysts and long‐term chemotherapy with benzimidazole derivatives such as albendazole or mebendazole [4]. Currently, several scolicidal agents including hypertonic saline (20%), silver nitrate (0.5%), ethanol (95%), cetrimide (0.1%), and chlorhexidine are used. However, each has limitations such as severe tissue necrosis, sclerosing cholangitis, peritoneal adhesions, risks of acute toxicity and anaphylaxis, hemolysis and local irritation [5, 6]. These complications underscore the urgent need for safer, more biocompatible alternatives. However, these approaches are limited by risks of cyst rupture leading to anaphylaxis or secondary hydatidosis, surgical complications, prolonged treatment duration, adverse effects (e.g., hepatotoxicity, leukopenia, alopecia), variable efficacy, and high recurrence rates [7]. These shortcomings highlight the urgent need for safer, more effective, and accessible alternatives, particularly scolicidal agents that can be used intraoperatively to inactivate protoscoleces and prevent dissemination [4].

Natural products, especially medicinal plants with established antiparasitic properties, have gained attention as promising sources for novel protoscolicidals [2, 8–10]. *Syzygium aromaticum* (clove), rich in eugenol, which may reach to 85%–95% concentration of clove essential oil, and other bioactive phenolics [11], exhibits broad‐spectrum antimicrobial, antifungal, and antiparasitic activities, including documented protoscolicidal effects against *E. granulosus* protoscoleces [12]. Despite its potential, challenges such as poor aqueous solubility, low bioavailability, volatility, and limited stability hinder clinical translation [13, 14]. Nanotechnology offers innovative solutions through advanced drug delivery systems like solid lipid nanoparticles (SLNs), which provide controlled release, enhanced solubility, improved bioavailability, reduced toxicity, and targeted action. The SLNs have shown promise in antiparasitic applications by overcoming limitations of conventional formulations [10, 15]. SLNs were selected over nanoemulsions and liposomes because of their superior physical stability, higher drug loading capacity for hydrophobic compounds like herbal oil, and the ability to provide sustained release. In addition, SLNs are also composed of biocompatible and biodegradable lipids, which decrease the risk of chronic toxicity compared with polymers. Since construction of SLNs is simple and more cost‐effective, large‐scale production and potential clinical translation are rationale [13]. It was suggested that employing SLNs as a carrier for albendazole not only significantly enhances the drug′s preventive efficacy against CE but also reduces its side effects [16].

Therefore, it seems that encapsulating clove oil (ClO) into SLNs could potentially enhance its stability, prolong its release, and reduce its systemic toxicity. The present study investigates the protoscolicidal efficacy of ClO‐SLNs against *E. granulosus* protoscoleces in an ex vivo model, alongside evaluation of cytotoxicity on HepG2 cells via MTT assay. This study was performed on HepG2 cell, which is a hepatic cell line. The main reason for selecting this cell line backs to this fact that the liver is the main organ affected by the CE. In addition, since hepatocytes are mainly affected by anti‐HC drugs, we selected HepG2 cell line to evaluate cytotoxicity effects due to ClO‐SLNs. This study is aimed at elucidating the potential of ClO‐SLNs as a safe and efficient protoscolicidal agent for adjunctive use in CE management, supporting future in vivo and clinical studies.

## 2. Materials and Methods

### 2.1. Preparation of ClO‐SLNs

Clove buds (*S. aromaticum*) oil was purchased (Lavender odor, Iran). To formulate ClO‐SLNs, emulsification‐probe sonication was hired [17]. Briefly, the lipid phase consisted of 1 mL of ClO, 800 *μ*L tween 80, 100 mg lecithin (Merck, Darmstadt, Germany), and 200 mg cholesterol (Sigma‐Aldrich) dissolved in 10 mL dichloromethane (Merck, Darmstadt, Germany) solvent. The resulting primary lipid phase was combined with 10 ml of 4% w/v PVA (Merck, Darmstadt, Germany) (organic phase: aqueous phase 1:1) and using an ultrasound probe sonicator (The ultrasonic processor UP400; Hielscher, Germany) at sonication cycle of 15‐s pulse by 10‐s pause, the mixture was homogenized at continuous 15000 Hz for 10 min. Subsequently, the resulting white cloudy emulsion was subjected to a Rota evaporator at 35°C for evaporation of dichloromethane [17]. The final nanoparticle yield was approximately 82%.

### 2.2. Physicochemical Characterization

A transmission electron microscope (TEM; EM10C‐100 KV; Carl Zeiss, Germany) was employed to investigate morphology of the nanoparticles as previously described [17]. Characterization of nanoparticles was carried out by dynamic light scattering (DLS) using Malvern particle size analyzer (Zetasizer 1033439, Malvern Instrument, United Kingdom) at 25°C with a detection angle of 173° (backscatter). Samples were diluted with deionized water and equilibrated for 2 min before measurement. The average particle size was calculated using Zetasizer Ver. 6.01 software and measured using a Zetasizer 1033439 (Malvern Instrument, United Kingdom). Data were presented as mean ± standard deviation (SD). Encapsulation efficiency (EE%) and drug loading (DL%) of ClO‐SLNs were determined by indirect method. Briefly, unencapsulated ClO was separated from nanoparticles by ultracentrifugation (20,000 rpm, 30 min, 4°C). The colloidal stability of ClO‐SLNs was evaluated by monitoring particle size and PDI over 30 days of storage at 4°C. The concentration of free ClO in the supernatant was measured spectrophotometrically at 280 nm. EE% and DL% were calculated using the following equations:

EE%=Total oil–free oilTotal oil×100.

DL %=Total oil–free oilTotal lipid+total oil×100.

Stock solutions were prepared under sterile conditions in a laminar flow hood. For clove ethanolic extract and ClO‐SLNs, serial dilutions were made from a 200 mg/mL stock in DMEM (without antibiotics) to achieve final test concentrations of 200, 100, 50, 10, 1 mg/mL, and 100 *μ*g/mL. ClO was diluted directly in DMEM to prepare the raw oil formulation. Albendazole was dissolved in DMSO as < 0.1% of final concentration and diluted to 10 mg/mL working concentrations. All solutions were filter‐sterilized (0.22 *μ*m) before use.

### 2.3. Collection and Isolation of Protoscoleces

Hydatid cysts were obtained from one naturally infected sheep liver at a local abattoir and transported on ice to the laboratory under sterile conditions. Approximately four cysts were surface‐sterilized with 70% ethanol, rinsed with normal saline, and aspirated using sterile syringes. Protoscoleces were isolated from cyst fluid, washed twice with normal saline, and their viability confirmed by eosin exclusion test (1% eosin; viable protoscoleces remain unstained, dead one′s stain pink). Only batches with ≥ 95% viability were used [18].

### 2.4. Cell Culture and Cytotoxicity Assay

The human hepatocellular carcinoma cell line HepG2 was cultured in DMEM supplemented with 10% heat‐inactivated fetal bovine serum (FBS) and 1% penicillin‐streptomycin, maintained at 37°C in a 5% CO_2_ humidified incubator. Cells were seeded at 30,000 cells/well in 96‐well plates and allowed to adhere for 24 h. Formulations were added at the indicated concentrations (in duplicate) and incubated for 48 h. Cytotoxicity was assessed using the MTT assay as follows: firstly, a 5 mg/mL stock of MTT (3‐(4,5‐dimethylthiazol‐2‐yl)‐2, 5‐diphenyltetrazolium bromide) solution (10X; 5 mg/mL in PBS) was prepared. Afterwards, 20 *μ*L of stock was added per well containing 180 *μ*L of cell culture medium (final volume 200 *μ*L) to reach a 1X solution, incubated for 4 h, and to solubilize formazan crystals, 200 *μ*L DMSO was added and plate was gently shaken for 20 min. Absorbance was measured at 570 nm using a microplate reader. Cell viability was calculated as a percentage relative to untreated controls [17].

### 2.5. Protoscolicidal Assay

Approximately 10,000 viable protoscoleces were exposed to each formulation concentration in microcentrifuge tubes (in duplicate) at 37°C. Time points were 15, 30, 60, and 120 min. At each interval, aliquots were stained with 1% eosin and examined under a light microscope (at least 300 protoscoleces counted per replicate). Mortality (%) was calculated as follows:

Mortality %=Number of stained protoscolecesTotal protoscoleces counted×100.

Albendazole served as positive control; untreated protoscoleces in DMEM were negative controls. Counting of protoscoleses was performed by two blinded investigators to reduce probable biases.

### 2.6. Statistical Analysis

Data are presented as mean ± standard error. Differences were analyzed using one‐way or repeated‐measures ANOVA followed by post hoc tests where appropriate. A *p* < 0.05 was considered significant. Statistical analyses were conducted using IBM SPSS Statistics Version 27.0 (IBM Corp., Armonk, New York, United States) and GraphPad Prism software, Version 8.3.0.538. All biological experiments (cytotoxicity and protoscolicidal assays) were repeated in duplicate and in three independent biological replicates.

## 3. Results

### 3.1. Electron Microscopy and ClO‐SLNs Characterization

**Figure 1:**
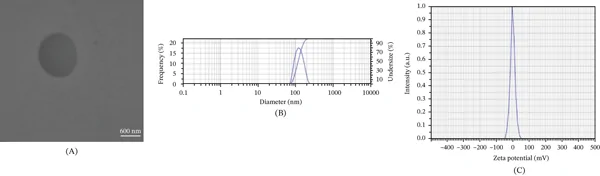
(A) The analysis of TEM images of ClO‐SLNs. The scale bar is 600 nm. (B) The DLS analysis of nanoparticles suggests a particle size 132.3 ± 30 nm with PDI of 0.37. (C) The zeta potential analysis showed a value of −0.9 mV.

After successful synthesize of ClO‐SLNs, TEM characterization showed round smooth margin particles (Figure 1A). The analysis of DLS showed a clear peak for SLNs sized 132.3 ± 30.0 nm with PDI of 0.37 (Figure 1B), whereas the zeta potential results showed −0.9 mV (Figure 1C). The ClO‐SLNs exhibited an encapsulation efficiency and a drug loading of 78.3*%* ± 4.2*%* and 12.7*%* ± 1.8*%*, respectively. The nanoparticles showed no significant changes in particle size or PDI (0.37 at Day 0 vs. 0.41 at Day 30), indicating acceptable short‐term stability. The EE % and DL were calculated for ClO‐SLNs that were 59.68% and 16.08%, respectively.

### 3.2. Cytotoxicity on HepG2 Cells (MTT Assay)

**Figure 2:**
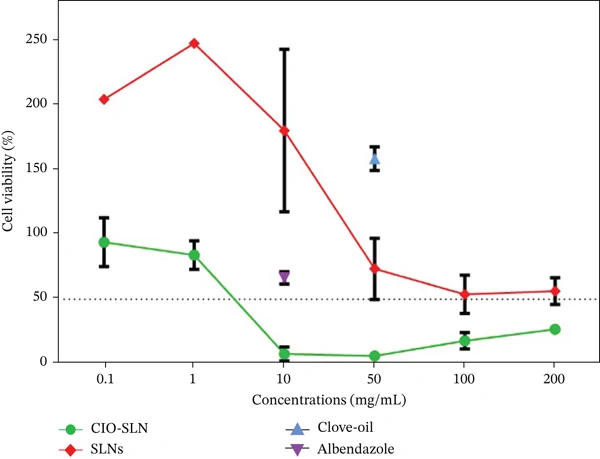
Cell toxicity assay of ClO‐SLN (green) and SLNs (red) on HepG2 cell line together with single concentrations of clove oil (blue) and albendazole (purple). No statistically significant reduction in cell viability was observed regarding the concentration (*p* value = 0.057). All tests were performed in duplicate and one‐way ANOVA was employed to analyze data.

The cytotoxic effects of the tested formulations on HepG2 cells were evaluated after 48 h of exposure using the MTT assay. The cell viability remained high (> 80%) across two concentrations of ClO‐SLNs (94.26*%* ± 18.91*%* for 100 *μ*g/mL and 84.31*%* ± 11.14*%* for 1 mg/mL), with no significant reduction (*p* = 0.057). The cell viability of SLNs in all concentrations remained > 50% with significant changes regarding concentrations (*p* value < 0.05). Raw ClO extract exhibited no cytotoxicity at 50 mg/mL and the cell viability of albendazole was 66.59*%* ± 4.81*%* at 10 mg/mL. These results indicate that ClO‐SLNs possess favorable biocompatibility and low cytotoxicity at protoscolicidal concentrations (Figure 2).

### 3.3. Protoscolicidal Activity

**Figure 3:**
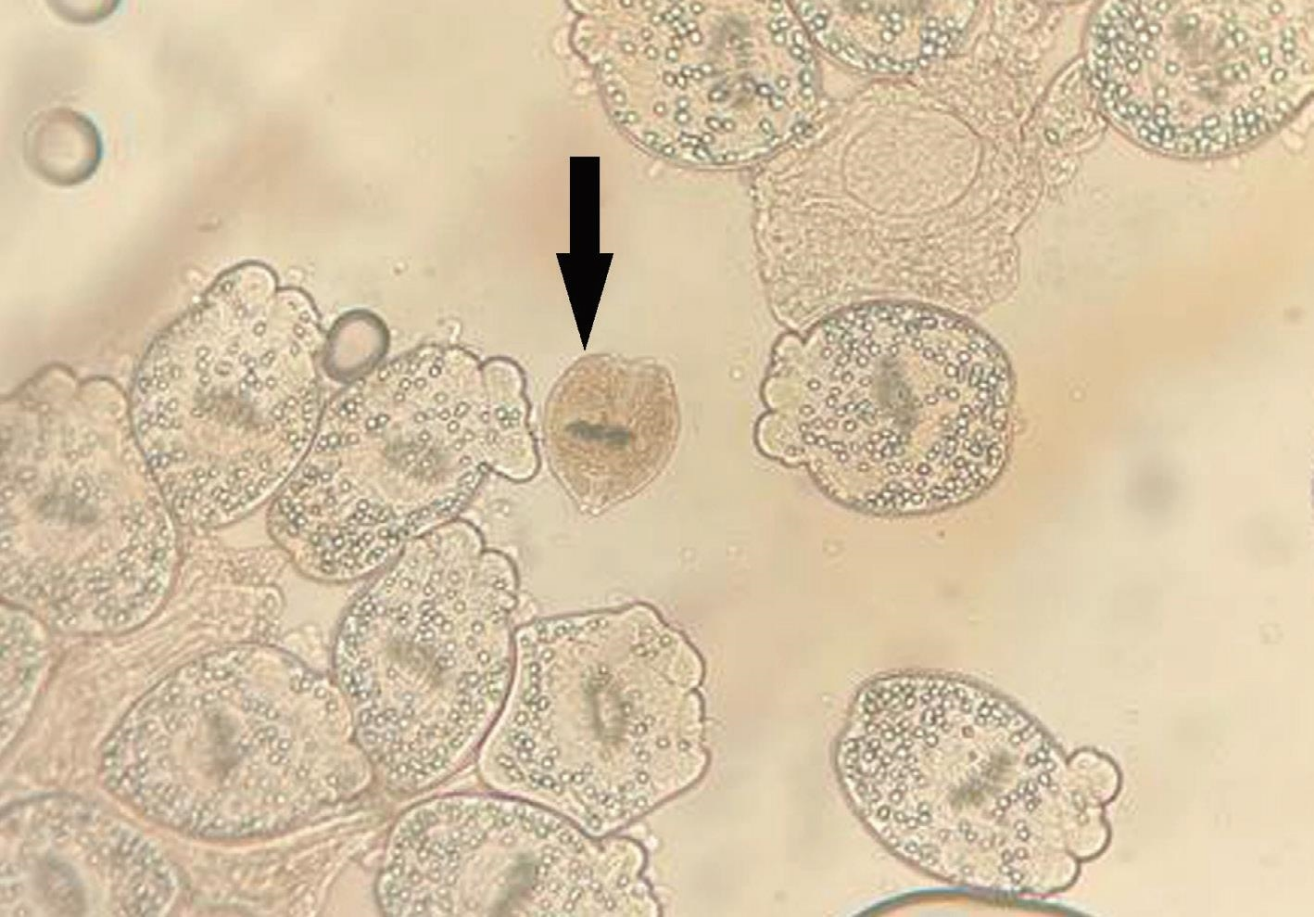
Protoscoleces are stained with eosin. The alive parasites remained unstained and dead protoscolex is indicated by black arrow.

**Figure 4:**
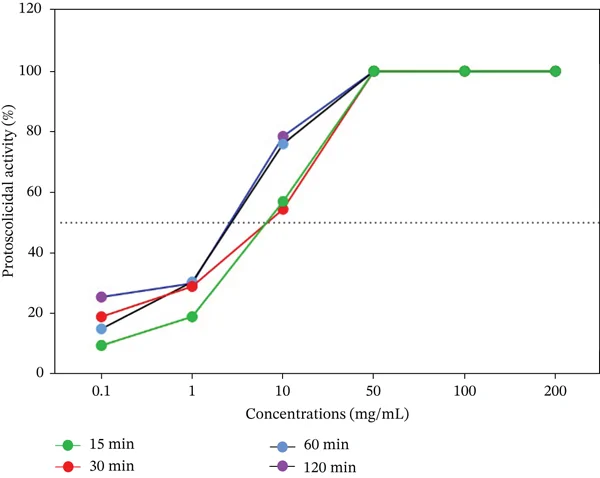
Protoscolicidal activity of ClO‐SLNs according to different concentrations in different time points. The mortality rates of protoscoleces exposed to ClO‐SLNs were concentration‐dependent in all time points with *p* values: 0.013, 0.0074, 0.0064, and 0.0042 for 15, 30, 60, and 120 min. All tests were performed in duplicate and one‐way ANOVA was employed to analyze data.

Dead protoscoleces were stained with eosin, whereas viable protoscoleces excluded the dye (Figure 3). The mortality rates of protoscoleces exposed to ClO‐SLNs were concentration‐dependent in all time points with *p* values: 0.013, 0.0074, 0.0064, and 0.0042 for 15, 30, 60, and 120 min, respectively (Figure 4). In the negative control group (DMEM alone), protoscolex viability remained > 95% throughout the 120‐min observation period. Albendazole (positive control) induced 100% mortality at concentrations of 10 mg/mL as early as 15 min postexposure, with no viable protoscoleces observed at subsequent time points (30, 60, and 120 min). The ClO‐SLNs exhibited rapid protoscolicidal effect among the tested formulations. In all time points, the mortality of protoscoleces was 100% in concentration ≥ 50 mg/mL. At concentration 10 mg/mL, high mortality, 76.00 ± 7.07 and 78.50 ± 3.54 was observed for 60 and 120 min, respectively, whereas during 15 and 30 min, the mortality was 57.00 ± 8.48 and 54.50 ± 7.78, respectively (*p* = 0.052). The concentration 1 mg/mL killed 19.00 ± 9.89, 29.00 ± 7.07, 30.50 ± 6.36, and 30.00 ± 8.48 from 15 to 120 min, respectively. The trend of protoscolicidal activity effects of ClO‐SLNs was not statistically significant (*p* = 0.510). The lowest mortality rates were observed in the concentration 0.1 mg/mL 9.50 ± 10.61, 19.00 ± 9.89, 15.00 ± 18.38, and 25.50 ± 12.02 from 15 to 120 min, respectively; however, a significant protoscolicidal activity was not seen based on the time‐points (*p* = 0.688).

## 4. Discussion

The results of the present study demonstrated that ClO‐SLNs exhibit potent scolicidal effects on protoscoleces of hydatid cysts, achieving 100% lethality at high concentrations (≥ 50 mg/mL) while maintaining low cytotoxicity (cell viability > 85%) on HepG2 hepatocytes. These findings underscore the superiority of ClO‐SLNs over raw ClO in terms of rapidity and potency, likely attributable to enhanced bioavailability, improved cellular penetration, and controlled release provided by the SLN matrix. Such attributes position ClO‐SLNs as a promising natural‐derived protoscolicidal agent for intraoperative use in hydatid cyst management, warranting further in vivo and preclinical evaluations.

The zeta potential of ClO‐SLNs was measured at −0.9 mV, which is lower than the ±30 mV threshold conventionally considered indicative of good colloidal stability. This finding warrants careful interpretation. Several factors may explain this observation. First, the presence of nonionic surfactants (Tween 80 and PVA) in the formulation can shield the surface charge and reduce the absolute zeta potential while still maintaining colloidal stability through steric stabilization mechanisms. Second, the zeta potential measurement is highly dependent on the dispersion medium′s ionic strength and pH; whereas our measurements were performed in deionized water, which may not fully reflect the stabilizing forces in biological media. Third, the lipid composition (cholesterol and lecithin) may contribute to a near‐neutral surface charge. Importantly, the absence of significant particle aggregation during the 30‐day stability assessment (as evidenced by stable particle size and PDI) confirms that the ClO‐SLNs maintain colloidal stability primarily through steric hindrance provided by the surfactant layer, rather than electrostatic repulsion. This phenomenon has been previously documented for SLN formulations stabilized with nonionic surfactants [19].

The findings of this study, along with the study by Machado et al., showed that the essential oil of *S. aromaticum* and its main compound eugenol exhibit strong antigiardial effects with IC_50_ values of 134 *μ*g/mL for the oil and 101 *μ*g/mL for eugenol, disrupting the growth, viability, adherence, and ultrastructure of *Giardia lamblia* [20]. These results are similar to the effects of ClO‐SLNs on hydatid protoscoleces, as both are based on ClO (containing eugenol) and induce comparable structural changes. Still, our nanoparticle form showed higher efficacy at lower concentrations. Similarly, another study investigated the number of different scolex‐stretching samples of *Holothuria leucospilota* extract and CeO_2_ nanoparticles for hydatid cyst protoscoleces and the results showed that they were reduced by 100% at 15 mg/mL for 60 min, increased caspase‐3, and reduced the number, size, and volume of cysts in infected mice [21]. This study aligns with our work, as both focus on using nanoparticles to enhance antiparasitic efficacy, but our ClO‐SLNs demonstrated lower toxicity on hepatic cells and could be a more natural option.

Eugenol is one of the main components of *S. aromaticum*. Antihydatid, antifibrotic, and immunomodulatory effects of *S. aromaticum* have been related to eugenol. In a mouse model of CE treated with eugenol oil and its nanoemulsion, it was reported that eugenol nanoemulsion reduced cyst weight, improved hepatic fibrosis, and increased IFN‐*γ* levels while decreasing IL‐4 [22]. Recent studies on water‐dispersible nanoparticles have demonstrated promising protoscolicidal activity against *E. granulosus* protoscoleces, providing a comparative perspective to our lipid‐based ClO‐SLNs. Rahimi et al. reported that biosynthesized silver nanoparticles (Ag‐NPs), originated from an aqueous extract of *Penicillium aculeatum* achieved 83%–90% mortality at concentrations of 0.1–0.15 mg/mL after 120 min of exposure, with concentration‐ and time‐dependent effects and ultrastructural damage to the tegument. In contrast, our clove‐SLNs exhibited superior rapidity and potency, attaining 100% lethality at ≥ 50 mg/mL within 15–30 min. This enhanced performance may be attributed to the lipid matrix of SLNs, which facilitates sustained release and deeper penetration into the lipophilic tegument of protoscoleces, compared with the burst release typical of water‐based metallic NPs [23]. Similarly, Aboelsoued et al. evaluated poly (amidoamine) (PAMAM) nanoemulsion, a hydrophilic polymeric system with high water dispersibility and positive zeta potential, which induced 100% mortality at 2 mg/mL within 20 min in vitro (and comparable ex vivo efficacy), accompanied by severe tegumental disruption including hook loss and membrane holes. Although PAMAM offers rapid electrostatic interactions with the negatively charged parasite surface, ClO‐SLNs achieved complete lethality in shorter times at higher but still effective concentrations, likely due to the controlled release and biocompatibility advantages of the SLNs carrier for hydrophobic eugenol‐rich ClO. Furthermore, unlike these water‐based systems, ClO‐SLNs demonstrated favorable hepatic cell viability (> 80% on HepG2), underscoring their potential as a safer, natural‐derived alternative for intraoperative protoscolicidal applications [24]. Our findings provide a robust foundation for advancing to clinical trials, demonstrating high protoscolicidal efficacy with minimal cytotoxicity, which could minimize risks like spillage during hydatid cyst surgery [9].

Despite the promising results of this study, several limitations should be acknowledged. First, all experiments were conducted in vitro, with ClO‐SLNs evaluated solely on isolated protoscoleces from hydatid cysts; thus, the findings cannot be directly extrapolated to in vivo conditions, where complex factors such as metabolism, biodistribution, host immune interactions, and intact cyst architecture may influence efficacy. Additionally, the lack of in vivo evaluation in animal models (e.g., secondary infection murine models of *E. granulosus*) represents a key limitation [25, 26]. Such studies are essential to assess cyst number, size, and volume reduction, hepatic fibrosis amelioration, cytokine modulation (e.g., increased IFN‐*γ* and decreased IL‐4), and overall safety profile [27]. Moreover, potential long‐term stability of ClO‐SLNs in biological fluids, possible immune responses in vivo, and optimal dosing regimens remain to be explored. Ultimately, further preclinical and clinical investigations are required to validate the potential of ClO‐SLNs as a protoscolicidal agent for intraoperative hydatid cyst management.

## 5. Limitations

In this study, there were some limitations. Firstly, the phytochemical composition of ClO was not evaluated using available techniques such as GC–MS analysis. In addition, this study was only performed on cell line, and an in vivo assay was not performed in suitable animal model. However, although this is a preliminary study, evaluating the current nano‐formulation may provide interesting data before clinical investigations.

## 6. Conclusion

In the present study, ClO‐SLNs exhibit cell viability above 80% across all tested concentrations with no significant cytotoxicity, while simultaneously displaying highly potent and rapid protoscolicidal activity against *E. granulosus* protoscoleces, achieving 100% mortality at concentrations ≥ 50 mg/mL across all time points. This study suggests ClO‐SLNs as a promising and effective protoscolicidal agent for intraoperative use in CE surgery, with strong potential to reduce the risk of secondary hydatidosis. Further in vivo before considering clinical evaluation are now warranted to confirm its therapeutic value and facilitate its translation into clinical practice.

## Author Contributions

H.M. and A.M. designed the study. Z.H., S.N., Z.M., and M.Z. provided natural products and synthesized nanoparticles. H.M. contributed to performing the experiments and analyzing the generated data. H.M. and A.M. contributed in writing the manuscript.

## Funding

This project was financially supported by the Shahid Beheshti University of Medical Sciences, Tehran, Iran with Grant Number: 43014068.

## Ethics Statement

All procedures performed in this study were in accordance with the ethical standards (IR.SBMU.RIGLD.REC.1403.048) released by the Ethical Review Committee of the Research Institute for Gastroenterology and Liver Diseases, Shahid Beheshti University of Medical Sciences, Tehran, Iran. As well, the study was approved by the ethics committee/institutional review board of the Alborz University of Medical Sciences (Ethic Code No. ABZUMS.REC.1404.175).

## Consent

The authors have nothing to report.

## Conflicts of Interest

The authors declare no conflicts of interest.

## Data Availability

Data sharing is not applicable to this article as no datasets were generated or analyzed during the current study.
